# The Role of Macrophages in Airway Disease Focusing on Porcine Reproductive and Respiratory Syndrome Virus and the Treatment with Antioxidant Nanoparticles

**DOI:** 10.3390/v16101563

**Published:** 2024-10-01

**Authors:** Kyuhyung Choi

**Affiliations:** 1 Department of Veterinary Pathology, College of Veterinary Medicine, Seoul National University, Seoul 08826, Republic of Korea; kyudac@snu.ac.kr; 2 Bundang New York Animal Hospital, Seongnam 13637, Republic of Korea

**Keywords:** ROS, PRRSV, respiratory disease, M1/M2, alveolar macrophages, nanomaterials

## Abstract

Lung macrophage cells play a critical role in various lung diseases, and their state can change depending on the progression of the disease by inducing either an inflammatory or anti-inflammatory state. In this review, the potential therapeutic effects of treatment with antioxidant nanoparticles in air-borne diseases focusing on porcine reproductive and respiratory virus (PRRSV), considering reactive oxygen species (ROS) as one of the factors that regulate M1 and M2 macrophages in the inflammatory and anti-inflammatory states, respectively, was described. In addition, the author examines the status of protein structure research on CD163 (one of the markers of anti-inflammatory M2 macrophages) in human and veterinary lung diseases.

## 1. Background

Lung disease has been one of the leading causes of death worldwide in humans for decades [1]. This applies not only to human medicine but also to the veterinary field. Infection with Distemper in canines [2], PRRS virus in porcines [3], and feline asthma [4] result in (cause) high prevalence and morbidity. To effectively diagnose and treat lung disease, it is important to understand its underlying pathogenesis mechanisms. Specifically, many researchers have recently addressed the role of macrophages in lung diseases. Some studies have focused on the correlation between changes in the state of macrophages, such as general M1/M2, classically activated macrophages, and alternatively activated and lung disease immunity, such as chronic obstructive pulmonary disease (COPD) [5,6]. Another theory centers on the role and correlation of alveolar macrophages and interstitial macrophages in various lung diseases [7,8].

In this paper, the progression of these M1/M2 theories is discussed.

## 2. Lung Epithelial Cells and the Status of Common Lung Diseases Associated with M1/M2

There are a variety of lung diseases, including pneumothorax, pneumonia, acute respiratory viral inflections such as influenza, COVID-19 etc., bacterial infections (tuberculosis, H. pneumonia and so on), and emphysema, bronchial asthma, COPD, and non-small-cell lung cancer are prevalent diseases that significantly harm public health, and much research is being conducted concerning both their pathogenesis and treatment. All chronic respiratory diseases have been reported to have a prevalence rate of 7% worldwide in 2017, with COPD (3.9%) and asthma (3.5%) accounting for the majority [9].

Lung epithelial cells play an important role in lung disease pathogenesis, and they include basal cells, secretory cells, type 1 pneumocytes, and type 2 pneumocytes. Additionally, alveolar macrophages and dendritic cells, along with epithelial cells, react to foreign antigens, and the former play an important role in the pathogenesis of asthma and COPD [10,11]. Harmful substances may also lead to macrophage stimulation, and along with lung epithelial cells, cytokines such as TNF (tumor necrosis factor)-a and IL (interleukin)-8 are secreted from epithelial cells, which induces the fibrosis of the bronchial tubes and alveolar macrophage destruction, resulting in irreversible airway obstruction [12]. In addition to exposure to various environmental allergens, cell signals in allergic asthma are mediated by the epigenetic regulation of histone protein methylation and acetylation in regulatory promoter nad enhancer regions of cytokine genes, resulting in enhanced cytokine gene expression such as IL-4 and IL-13 that induce the M2 polarization of macrophages [13]. Furthermore, cytokine TNF-a, lipopolysaccharide (LPS), and reactive oxygen species (ROS) also induce M1 polarization in acute lung injuries [14].

The M1 and M2 theories of macrophages were previously conceptualized by the Mantovani research team [15].

**Figure 1:**
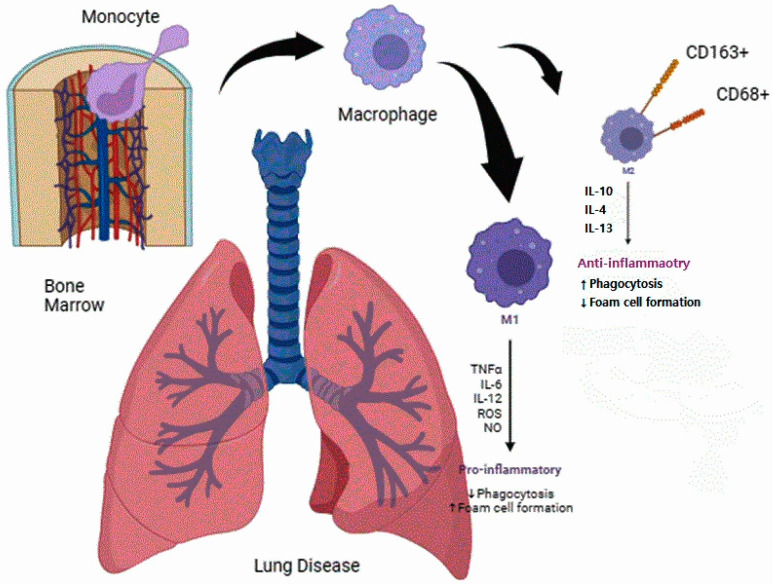
Alveolar macrophages, interstitial macrophages, and monocytes (differentiated from bone marrow). Scheme of bones in a lung-inflammatory state. Monocytes are differentiated in the bone marrow, and alveolar macrophages are differentiated into the M2 state to phagocytose pollutants and fight inflammation [18].

Macrophages are differentiated into M1 and M2 in inflammatory and anti-inflammatory states, respectively, depending on whether the immune response is Th1- or Th2-induced, and it has been revealed that ROS, among various cytokines, plays an important role in inducing an inflammatory state [16]. Tissue-stored macrophages can also be induced by cytokines and chemokines, depending on the inflammatory condition, and move into the blood vessels. When additional macrophages are needed, monocytes are differentiated into macrophages in the bone marrow. The states of these macrophages are also polarized from M0 to M1 or M2 to confront situations such as inflammation, cancer, and injury. They circulate within blood vessels and are stored in tissues to maintain balance and homeostasis between tissue-resident and circulating macrophages (Figure 1) [17].

Accordingly, researchers have used antioxidant treatments to alter macrophages to an anti-inflammatory state [19,20]. Furthermore, research has been conducted concerning the treatment of diseases through the anti-inflammatory state of macrophages [21]. Research is also being applied to cancers such as non-small-cell lung cancer [22].

### Alveolar Macrophages and Interstitial Macrophages

Macrophages are classified into alveolar and interstitial depending on which part of the lung they are located, and research has been continued to study the characteristics of these differentiated macrophages to reveal the pathogenesis of lung disease. Alveolar macrophages are known to adhere to and phagocytize antigens faster than interstitial macrophages. These two macrophages achieve homeostasis and change their state according to the inflammatory environment of the lung [7,23]. In this paper, the author focuses on the M1/M2 theories rather than alveolar/interstitial homeostasis.

## 3. Sequence–Structure–Function Theory of Antigens and the Application of Macrophage Epitopes in Various Diseases, Including Lung Disease

Previous research demonstrating that the amino acid sequence determines both the protein structure and the function of the protein molecule has become widely known, and, to date, research is being conducted to predict the structure of many proteins to diagnose or treat specific diseases [24]. For example, researchers recently discovered the structure of the coronavirus antigen’s spike protein, which was imitated to make the most recent COVID-19 vaccine [25]. This sequence–structure–function theory has also been used for other rare diseases, such as pheochromocytoma [26], which is not only rare but also fatal. Proteins such as Chromogranin A (CgA) have been used as biomarkers of pheochromocytoma as a result of previous research to reveal their sequence and structure [26]. Additionally, it is known that CD163 is overexpressed in COPD patients, and it may be related to the crucial pathogenesis of the disease [27].

These efforts have also been applied to macrophages to reveal the structure of macrophage CD163 in inflammatory situations and the mechanism of disease [28], and it is described in Section 4 as a GP2 and GP4 protein structure. In particular, research is actively underway concerning the pathogenesis of PRRSV, which is one of the most widespread respiratory diseases, causing tremendous economic loss worldwide [29].

## 4. Lung Disease: Focusing on PRRSV

Porcine reproductive and respiratory syndrome virus (PRRSV) is an agent of a mysterious pig disease that was first discovered in the United States and Canada in the late 1980s, causing respiratory diseases, miscarriages, and reproductive diseases such as infertility. In the 1990s, this disease was discovered in European countries such as the UK and France, and also in Korea, China, and Japan. In particular, the virus was isolated for the first time in Korea in 1994 [30]. Since then, PRRSV has caused serious economic damage all over the world and has existed through mutations such as the North American type, the European type, and highly pathogenic PRRSV.

**Figure 2:**
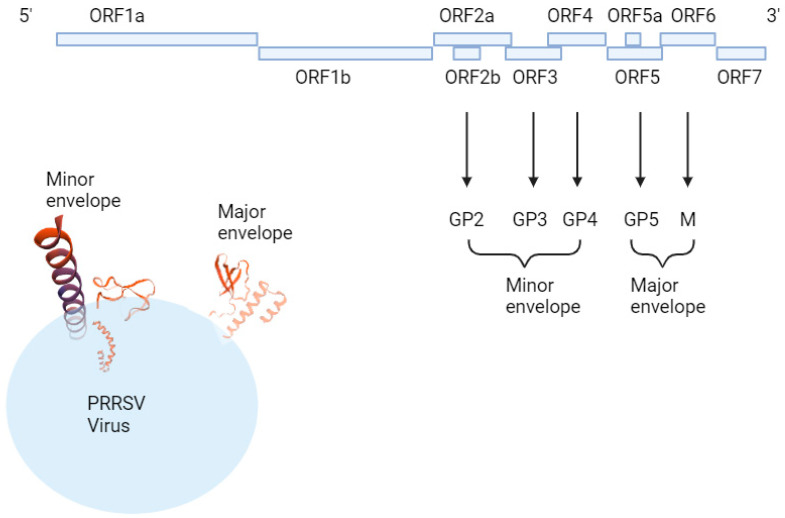
Schematic illustration of PRRSV structure made by SWISS-MODEL prediction program (https://swissmodel.expasy.org, accessed on 22 May 2024).

PRRSV is a small, single-stranded, positive-sense, enveloped RNA virus which belongs to the family Arteriviridae. Genomic RNA is 15 kb long and consists of a part that produces non-structural proteins (ORF 1a and ORF 1b) and structural proteins (ORF 2, ORF 3, ORF 4, ORF 5, ORF 6, and ORF 7). ORF 2, ORF 3, ORF 4, and ORF 5 produce GP 2, GP 3, GP 4, and GP 5, while ORF6 produces M protein, and ORF7 produces N protein [31]. PRRSV is genetically divided into two types, which are European type 1 and North American type 2 viruses. The most popular strains of each type are Lelystad, Netherlands [32], European strain type 1, VR-2332 [33], and North American strain type 2. Both the North American and European types are structured with a major envelope and minor envelope. The major envelope protein consists of M (174 residues in type 2 and 173 in type 1) and GP5 (200 residues in type 2 and 201 in type 1). Minor envelope protein GP2 (256 residues in type 2 and 253 in type 1), GP3 (254 residues in type 2 and 265 in type 1), and GP4 (178 residues in type 2 and 183 in type 1). M and GP5 form the domain structure, and GP2, 3, and 4 come together to form a functional structure [34] (Figure 2).

PRRSV mainly infiltrates the respiratory system of pigs and causes reproductive and respiratory symptoms, and the most important host cells at this time are alveolar macrophages in the lung. Among macrophage viral receptors, CD163 and sialoadhesin have been studied the most [34]. It is known that the macrophage CD163 marker interacts with viral GP2 and GP4, and the macrophage sialoadhesin marker interacts with GP5, but the specific interaction process has not been fully elucidated [34].

Interestingly, it has been revealed that porcine CD163 provides the infections of both European PRRSV (type 1) and North American PRRSV (type 2), whereas human CD163 supports only type 2, and whether type 1 is involved is not revealed. Also, CD163 Knock-Out swine is resistant to both type 1,2 PRRSV, whereas SRCR5-swap CD163 swine is resistant for only type 1, not type 2 [35]. This fact implies the domain of SRCR5 CD163 macrophage receptor and PRRSV minor envelope GP2,4 are crucial to elucidate the pathogenesis of PRRSV and different virulence of type 1 and type 2 that may be related to structural differences in the interaction site due to the residue difference between the two types.

There are various routes of infection for PRRSV, including respiratory, oral [36], transdermal, and needle injection [37]. When the virus enters the body through various infection routes, primary proliferation occurs in bronchial mucus, lung parenchymal cells, such as type 1, 2 pneumocyte, and macrophages. Within 12 hours, secondary proliferation occurs in regional lymph nodes and enters the blood leading to viremia. Afterwards, the systemic infection progresses and becomes a subclinical infection or presents various clinical symptoms such as breathing difficulties, neurological symptoms, and abortion. After recovery, the virus is shed through blood, stool, saliva, or semen through persistent infection [38].

When primary infection is caused by mixed infection with PRRSV, mycoplasma, and porcine circovirus (PCV), in combination with environmental stress such as contaminated gas and dust, the pathogen phagocytosis ability of macrophages and the immunity of bronchial cilia decrease, making it easier for pathogens to infiltrate and proliferate in the body. In addition, subsequent ? complex infection with secondary pathogens such as *Actinobacillus pleuropneumoniae*, *Pasteurella multocida*, and *Glaesserella parasuis* can lead to Porcine respiratory disease complex (PRDC) [39] and further worsen clinical signs.

Clinical symptoms differ slightly depending on the age of the infected pig. For example, for sows, abortion, premature birth, delivery of weak piglets, and stillbirth have been demonstrated [40]. Preweaning piglets have a high mortality rate of close to 100%, neurological symptoms, and respiratory difficulties. Poor growth and increased mortality in raised sows and piglets have also been noted. In finishing pigs, loss of appetite and high fever are possible symptoms, and in boars, sperm motility decreases, and high fevers are representative clinical signs [41].

In North America, such as Canada and the United States, North American infections dominate, while in European countries, such as Italy and Russia, European infections dominate. In particular, many European-type and North American-type complex infections are found in Asia, including Korea [42]. The highly pathogenic PRRSV, a North American variant, is mainly spread in North America, and in Southeast Asia, including Vietnam, the highly pathogenic PRRSV variant is also widespread [43]. The prevalence rate of PRRSV among continents is not well-documented, yet there have been many studies that elucidate the origin of the virus, both type 1 [44] and type 2 [45], using genetic epidemiology and phylogenetic tree. 

The virus can be excreted through blood, urine, feces, nasal juice, saliva, and semen, and orderly and persistently infected pigs can excrete the virus through pharyngeal mucus for up to 5 months [46]. The spread of the virus includes intra-farm and inter-farm infection. Infections within the farm include placental, oral, nasal, and genital infection; direct and indirect contact; flies; mosquitoes; air; and poor hygiene of worker infections. Farm-to-farm transmission includes infection through the movement of infected pigs, semen, and contaminated vehicles and equipment [36].

The diagnosis of PRRSV can be divided into antigen and antibody detection, which can be performed on blood samples, tissues, saliva, semen, etc. There are many types of diagnostic tests, immunohistochemistry (IHC), virus isolation, and in situ hybridization with labeled probed and reverse transcription with subsequent polymerase chain reaction (PCR) with TaqMan probes for detection in real time [47]. antibodies specific antibodies are detected by means of enzyme-linked immunosorbent assay (ELISA) or indirect fluorescent assay (IFA) [48].

Additionally, enzyme-linked immune absorbent spot (ELISPOT), which measures PRRSV-specific interferon gamma (IFN-γ) in serum, is commonly used at present [49].

For preventing PRRSV, it is necessary to block horizontal transmission through all-in, all-out strategy and biosecurity, and nursery depopulation, and improvement in breeding facilities are also crucial [50]. For monitoring the disease, conducting regular inspections, especially detailed inspections and serological tests for suspicious pigs are key to effectively protect the pig from PRRSV.

PRRSV displays complex interactions with the host immune system, including modulating innate immune responses and employing various immune evasion strategies. One aspect of this dynamic interplay is the involvement of macrophages, which are critical players in the host’s antiviral defense. In particular, alveolar macrophages are among the first cells to encounter PRRSV upon respiratory infection, along with type II pneumocyte [51]. When PRRSV encounters the macrophage, sialoadhesin, the macrophage-specific receptor is responsible for the internalization of the virus. Together with another macrophage marker, CD163, macrophages become more susceptible to PRRSV infection [52]. PRRSV not only replicates in the host macrophages, but they also induce apoptosis [53]. Then, the virus evades host humoral immunity and cell-mediated immunity which are proven by a decrease in neutralizing antibody and interferon (IFN) [54]. Interestingly, PRRSV also enters neuroendocrine cell which is shown by the biomarker chromogranin A [55], and it is prominent, especially in highly pathogenic PRRSV (HP-PRRSV), which is originated from type 2 PPRSV. The higher concentration of neuroendocrine biomarkers in the highly pathogenic PRRSV of North American origins rather than the European type means that the North American virus may have a higher ability to penetrate neuroendocrine organs when it infiltrates the porcine system [55]. The specific mechanism for this requires additional research.

Like previously described, the virus has evolved various strategies to disrupt the host’s antiviral systems and promote its own survival. A crucial aspect of these strategies involves the interaction between PRRSV and the host’s immune cells, particularly macrophages. PRRSV has been shown to preferentially infect and replicate within M1 and M2 macrophage subsets, with distinct effects on their functions. For example, type 2 PRRSV can induce the M1 polarization of macrophages and Th1 response, which are shown by the host’s cell surface receptors, CD163, and proinflammatory genes, IFN-γ and IL-12 [56]. The exact mechanism of this process is not fully elucidated, but a follow-up study is still ongoing. 

Importantly, PRRSV infection can also modulate the polarization of macrophages, skewing them towards an M2-like phenotype. Since the M2 type induces a macrophage response related to anti-inflammation, the M2 state may be more advantageous for PRRSV in host invasion, replication, and survival than the inflammatory M1 macrophage state. As briefly described previously, PRRSV regulates the M1 and M2 differentiation of macrophages, thereby regulating the immune response and facilitating virus invasion [57]. If this control can be artificially controlled in the opposite direction of PRRSV through drug administration, a more efficient vaccine effect can be expected. Additionally, this macrophage differentiation strategy has the potential to be applied not only to PRRSV but also to other lung diseases. Some studies have shown that treatment with nanoparticles consisting of antioxidants can provide an antioxidant effect, and it has been shown via in vitro and in vivo experiments [58,59]. Through this antioxidant activity, reactive oxygen species (ROS) can be removed, which is essential chemokine for M1 polarization, so that the state of macrophages can be eventually changed from the M1 state to the M2 state. Therefore, if an appropriate nanoparticle candidate is selected to scavenge ROS, it is possible to develop a lung disease medicine or adjuvant that is harmless to the body. Among the many candidates, selenium and magnesium, which are essential trace elements in the body, are harmless to the body and show antioxidant activity comparable to that of heavy metals such as silver or gold [60,61]. If such nanomaterial synthesis research is conducted along with macrophage differentiation, it will not only provide more effective PRRSV defense but also be applicable to various lung diseases. This could be a promising strategy because, among various infectious diseases such as human influenza, treating the disease with nanoparticles has been successful a decade ago [62], and it has also been applied to animal diseases in the veterinary field at present [63]. Along with studies that elucidate the structural interaction of the CD 163 macrophage receptor, treating nanomaterials to control macrophage polarization can provide a key factor in preventing lung diseases, including PRRSV.

## 5. Therapeutic Effects of Changes in Macrophage Status through ROS Scavenging through Nanoparticles

Currently, various researchers, such as Korea’s Hyeon Taeghwan group and China’s Zhiyuan Zhong group, have conducted studies on the therapeutic effects of macrophage polarization in treating inflammatory diseases with nanoparticles. Successful research results have been achieved in the fields of Crohn’s disease IBD [64], sepsis [65], and rheumatism [66] using ROS scavenging.

Research has also been conducted on the role and differentiation of macrophages in various lung diseases, such as COPD [67], non-small-cell lung cancer [68], and acute/chronic inflammatory lung disease [69]. In particular, in non-small-cell lung cancer, the expression of CD68 and the M2 marker CD163 increased, whereas that of iNOS (the M1 marker) decreased [70]. 

Combining the concept of macrophage modulation through nanoparticles and the role of macrophages in lung diseases, the treatment of lung diseases is also progressing through macrophage modulation using nanoparticles [71]. However, this research has been conducted recently compared to other diseases. The goal of inducing macrophage differentiation differs depending on both the inflammatory and cancer conditions. M2 are anti-inflammatory macrophages that promote tumor development and metastasis, whereas M1 are proinflammatory macrophages that suppress tumor development and metastasis [72].

**Figure 3:**
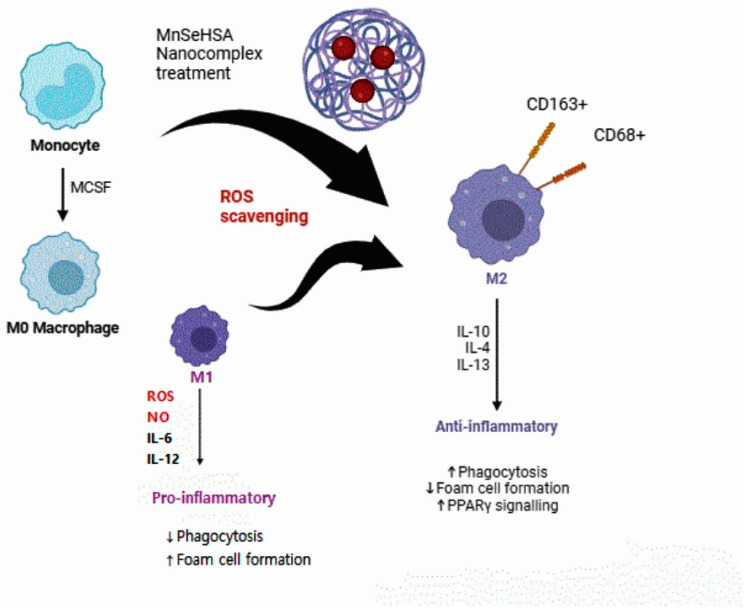
The nanocomposite (MnSeHSA) for treatment of inflammation by altering the state of macrophages from M1 and M0 to M2 (which is anti-inflammatory).

When synthesizing nanoparticles using the method published by Yang in 2017 [73], (using selenium and manganese, which are essential trace elements, instead of ceria, which is a heavy metal), comparable ROS scavenging ability was found. During the preliminary tests, selenium–albumin and manganese–albumin nanocomposites exhibited excellent antioxidative effects in vitro, as did ceria–albumin nanomaterials. Although more experiments and research should be conducted, including in vivo tests to apply this material, follow-up research can be conducted to investigate the therapeutic effects of lung disease inflammation (Figure 3). This strategy would not be suitable for lung cancer diseases, as inducing the M2 phenotype promotes tumor progression. It is also known that M2 macrophages cause a significant rise in asthma [74], COPD [75], and lung fibrosis [76]. However, the ROS scavenging strategy focuses on recovering M1/M2 homeostasis under bias towards the M1 population environment. Therefore, the mouse model incorporating this Se/Mn/albumin nanomaterial that is recommended for in vivo testing must consider macrophages in the M1 state that are dominant in severe and critical lung diseases that cause sepsis such as COVID-19 [25].

### Effects of Nanoparticle Treatment on the Human Body

When treating with nanoparticles, these could be deposited into the bone marrow, spleen, and central nervous system (CNS), which is a critical issue because the spleen is an organ where various immune cells are activated. We also have to consider the accumulation of nanoparticles in the human body when it is applied to PRRSV because of the characteristics of economic food animals. If heavy metal nanomaterials are deposited in the spleen, bone marrow, or CNS, there may be unexpected side effects, which may cause more harm than good in the treatment. According to a past study, a nanomaterial-bound anticancer drug showed higher bone marrow toxicity than a nanomaterial-free anticancer drug [77]. Therefore, researchers have tried to treat cancer or related diseases with nanomaterials while minimizing the impact on the spleen and bone marrow by directly delivering the nanoparticles to the target organ using other cells such as nanoparticle-attached red blood cells [78]. Nanoparticlescan entry into eukaryotic cells by endocytosis with their endosomal-lysosomal entrapment with hydrolysis with lysosomal enzymes. The spleen is responsible for facilitating absorption into the body and removing blood cells through macrophages. Hepatic circulation, splenic uptake, and elimination of the nanoparticles are the critical parts of biodistribution [79,80]. Moreover, the spleen is an organ responsible for immunity in our body, and it contains macrophages in various states [81]. Depending on external stimulation, it is differentiated into the M1 or M2 state [82]. Spleen-derived macrophages play a protective role in lung inflammation [83]. Additionally, some studies have found decreased responsiveness in patients with interstitial lung disease due to the splenic reuptake of the drug [84]. Considering these points together, when treating lung disease using nanoparticles, it is imperative to consider side effects due to nanoparticle absorption in the body and reuptake in the spleen, in addition to maintaining M1/M2 balance through the ROS scavenging strategy. This further supports the fact that nanoparticles should be synthesized not with heavy metals but with essential trace elements such as selenium and manganese, which can minimize side effects, and albumin, which is a basic component of cells but has antioxidant properties. Various studies have also found selenium to be hepatoprotective against heavy metal toxicity [85,86]. Albumin treatment is beneficial as a natural lung surfactant [87], and it was shown that inhalable albumin-based nanoparticles exhibited excellent potency in lung cancer and was drug-resistant [18]. This suggests that, instead of commonly used heavy metal nanoparticles, including silver, gold, and ceria, the former selenium–human albumin-based nanoparticle inhalant is an excellent candidate in treating lung diseases such as COVID-19 or sepsis-induced lung damage.

## 6. Macrophages and Lung Diseases in the Veterinary Field

Therefore, macrophages play an important role in the pathogenesis of lung diseases of various species, and, in particular, research into M2 has been conducted by altering the state of macrophages from M1 to M2 through studies on the protein structure and function of CD163 and through treatment with nanoparticles to prevent disease.

**Figure 4:**
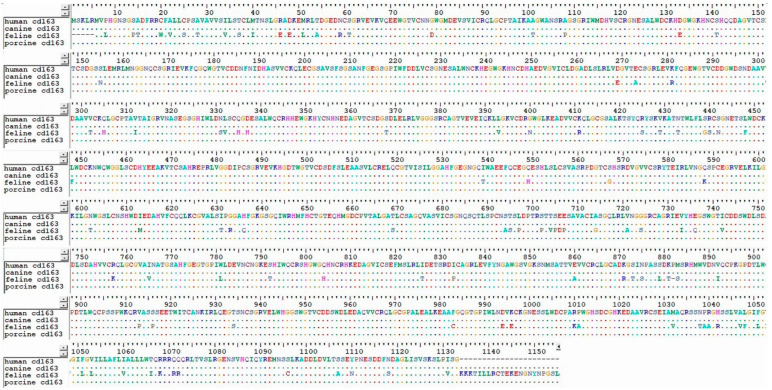
Amino acid sequence alignment of human, canine, feline, and porcine macrophage CD163.

In PRRSV, scavenging ROS through selenium–albumin nanomaterial treatment can lead to the polarization of macrophages into the M1 state to M2 state, which express anti-inflammatory cytokines such as IL-10, IL-4, and IL-13 result in the upregulation of phagocytosis and PPAR signaling (Figure 3). The suggested treatments have caught the attention of many scientists in this field. Relevant research in the veterinary field is also in progress. For example, one study discovered that the CD163+ alveolar macrophages of sheep were latently infected with the ovine progressive pneumonia (OPP) virus antigens. However, the direct role of macrophages in the infection mechanism has not yet been revealed. Furthermore, the role of porcine alveolar macrophage in the PRRS infection has not yet been fully elucidated. It was also structurally revealed that CD163, especially the SRCR5 receptor, plays a critical role [88]. Accordingly, the author explored areas in which additional research could be conducted in the veterinary field and compared the amino acid sequences of CD163 macrophages from humans, dogs, cats, and pigs (Figure 4). As a result, except for cats, humans, dogs, and pigs had almost identical amino acid sequences. Because the sequence of CD163 slightly differs in cats, there may be differences when applying the existing M1 and M2 theories to lung disease. Limited research has been conducted on cat CD163 and the infection mechanism of lung disease. If research on this progresses, new treatments may be developed for intractable lung diseases such as asthma, latent herpes infection, and chylothorax.

## 7. Conclusions and Perspectives

In this paper, we briefly reviewed the types of lung diseases in the human medicine and veterinary fields, the role and differentiation status of macrophages in lung diseases, the status of the treatment of various diseases using macrophages, and nanomaterials that can be applied to lung diseases. In particular, macrophages play an important role in the pathogenesis of lung diseases of various species, and nanomaterials that consist of trace essential elements can scavenge ROS without damage or accumulation to the host body and induce M2 polarization from the M1 state. It can be utilized into the treatment or other lung disease such as PRRSV. Furthermore, the role of porcine AM in the PRRSV infection has not yet been fully elucidated. It was also structurally revealed that CD163, especially the SRCR5 receptor, plays a critical role in the interaction with GP2 and GP4 of epitopes. Along with the M1/M2 strategy, a better understanding of the functional domain could be the key to elucidate this interaction mechanism and will provide efficient protection of the disease. In human medicine, M1/M2 theories can be applied to lung inflammatory diseases by treating nanoparticles to polarize macrophages to M2 through ROS scavenging, especially selenium–manganese albumin nanomaterials, which are powerful antioxidants that are harmless to the human body. This study also addresses the importance of the impact of nanoparticles on human body organs such splenic reuptake and bone marrow deposition toxicity. In veterinary medicine, M1/M2 theories could be applied to porcine PRRS virus infection and other animal lung diseases, such as the sheep OPP virus. Finally, it suggests the need for follow-up research to explore the role of macrophages in various feline lung diseases through the sequence–structure–function theory via structural research on feline CD163.

## Abbreviations

ROS (reactive oxygen species), AM (alveolar macrophage), IM (interstitial macrophage), PRRSV (porcine reproductive and respiratory syndrome virus).
